# Breast Cancer Genes PSMC3IP and EPSTI1 Play a Role in Apoptosis Regulation

**DOI:** 10.1371/journal.pone.0115352

**Published:** 2015-01-15

**Authors:** Eva Capdevila-Busquets, Nahuai Badiola, Rodrigo Arroyo, Víctor Alcalde, Montserrat Soler-López, Patrick Aloy

**Affiliations:** 1 Joint IRB-BSC-CRG Program in Computational Biology, Institute for Research in Biomedicine (IRB Barcelona), Barcelona, Catalonia, Spain; 2 Institució Catalana de Recerca i Estudis Avançats (ICREA), Barcelona, Catalonia, Spain

## Abstract

A key element to delineate the biology of individual tumors is the regulation of apoptosis. In this work, we functionally characterize two breast cancer associated genes, the proteasome 26S subunit ATPase 3 interacting protein (PSMC3IP) and the epithelial-stromal interaction 1 (EPSTI1), to explore their potential apoptotic role in breast cancer. We first explore the existence of direct physical interactions with annotated BC-apoptotic genes. Based on the generated interaction network, we examine several apoptotic markers to determine the effect of PSMC3IP and EPSTI1 gene expression modulation in two different human breast cancer cell lines to suggest potential molecular mechanisms to unveil their role in the disease. Our results show that PSMC3IP and EPSTI1 are able to modulate the extrinsic apoptotic pathway in estrogen receptor positive and triple negative breast cancer cell lines, highlighting them as potential therapeutic targets.

## Introduction

Due to its complexity, breast cancer (BC) is often considered a broad set of diseases including multiple, distinct biological subtypes with diverse natural histories that present a varied spectrum of clinical, pathological and molecular features with different prognostic and therapeutic implications [1]. The poor prognostic outcome of breast cancer is largely due to its resistance to current cancer therapies, where the balance between cell proliferation and apoptosis plays a critical role and it is crucial in determining the overall growth or regression of the tumor in response to treatments [2]. Hence, identifying proteins involved in apoptosis resistance developed by tumorigenic cells has an essential importance in order to find new therapeutic approaches. Two major apoptosis pathways have been described: the mitochondria mediated (intrinsic) and the death receptor mediated (extrinsic), which is initiated by the binding of ligands such as TNF-α or TNF-related apoptosis inducing ligand (TRAIL) to death receptors [3]. Once the receptors are activated, they oligomerize and form complexes that recruit and activate the initiator caspase-8. Active caspase-8 subsequently cleaves effector caspases like caspase-3 and caspase-7, resulting in activation or inactivation (as well as translocation), of several substrates such as the poly ADP ribose polymerase (PARP), with the consequent induction of cell death [4, 5].

In the last decade, network biology approaches have contributed to identify novel causative and susceptibility oncogenes, as well as secondary effectors that could not be highlighted by conventional analysis based on differential expression [6] Therefore, this novel approach can provide a deeper understanding of the molecular mechanisms underlying complex pathological processes, offering new biomarkers that may help to improve breast cancer diagnosis.

*PSMC3IP* gene is located on chromosome 17q21, proximal to *BRCA1* [7] and previously linked to breast cancer predisposition [8]. It has been characterized as a nuclear receptor participating in estrogen, androgen, glucocorticoid and progesterone receptor–mediated gene regulation [9, 10]. PSMC3IP is upregulated in breast cancer [7, 11] and in addition, inactivating mutations [12] have also been shown to regulate DNA recombination in DNA repair [13], potentially contributing to an increased risk in familial breast and ovarian cancers.

On the other hand, *EPSTI1*, an interferon (IFN) response gene [14], has been identified as a stromal fibroblast-induced gene in breast cancer, being highly upregulated in invasive breast carcinomas as compared with normal breast [15, 16]. More recently, it has been shown that expression levels of *EPSTI1* associate with tumor initiation and migration, stem cell–like properties, epithelial-mesenchymal transition (EMT) [17] and breast cancer invasion and metastasis [18], with the highest expression observed in basal-like subtype breast cancer cells exhibiting a poor prognosis [17].

Although the relationship of PSMC3IP and EPSTI1 with BC is well established, the underlying molecular mechanisms are still unknown. In the present study, we describe novel interactions between PSMC3IP and EPSTI1 with well-established BC genes which are also related to apoptosis and cell proliferation processes. We explore the anti-apoptotic role of PSMC3IP and EPSTI1 and their contribution in breast cancer development. We have carried out a functional characterization associated to cell apoptosis by means of caspase-8 and-3 activation, PARP cleavage and DNA integrity, based on gene overexpression and silencing in two different human breast cancer derived cell lines under both basal and apoptotic-induced conditions.

## Materials and Methods

### Subcloning of human cDNAs into Y2H plasmids

Human ORF clones were cloned into pENTR⁄D-TOPO vector (pENTR Directional TOPO cloning kit; Life Technologies) and sequence verified. AKT1, BCAR3 and EPSTI1 clones derived from the human ORFeome v1.1 [19]; CASP8 and PSMC3IP from Life Technologies Ultimate ORF Clones [20]. All ORFs were individually transferred into yeast two-hybrid (Y2H) destination vectors by Gateway recombinational cloning (ProQuest Two-Hybrid System, Life Technologies). BC-apoptosis genes were cloned into pDEST32 to generate bait plasmids and the BC associated genes (EPSTI1 and PSMC3IP) were cloned into pDEST22 to obtain prey plasmids.

### Y2H matrix screens

Bait and prey plasmids were pair-wise co-transformed into MaV203 yeast strain in 96-well arrays and plated onto selective SD2 (lacking Leu and Trp amino acids) agar media and incubated for 48 hours at 30°C to detect colony growth. Co-transformant arrays were then replica plated onto different selective media for interaction screening. To assay the activation of the HIS3 reporter gene, SD3 (lacking Leu, Trp, His) agar plates were supplemented with 12 to 100 mM of 3-aminotriazole (3AT, Sigma-Aldrich), being 50 mM 3AT the optimal concentration for positive HIS3 activation colonies. Similarly, we assayed the activation of the URA3 reporter gene by plating onto SD3 (lacking Leu, Trp, Uracil). Double reporter HIS3/URA3 activation was evaluated by SD4 (lacking Leu, Trp, His, Uracil) agar plates supplemented with 20 mM of 3AT and LacZ reporter gene was tested by the β-galactosidase assay on a nylon membrane placed onto a SD2 agar plate.

In order to minimize the number of false positives (at the cost of penalizing potential false negatives as well), we subsequently scored the positive interactions based on their ability to activate at least two reporter genes or being repeatedly observed in biological replica screens, which defined our high-confidence (HC) interaction set.

### Cell culture

MDA-MB-231 and MCF-7 cells were kindly provided by Dr. Violeta Serra (VHIO, Barcelona, Spain). MDA-MB-231 were cultured in Dulbecco’s Modified Eagle Medium (DMEM)/F12 and MCF-7 were maintained in Dulbecco’s Modified Eagle Medium (DMEM) (GIBCO Life Technologies), both supplemented with 10% Fetal Bovine Serum (FBS) and 1% Penicillin-Streptomycin (10,000 U/mL) purchased from GIBCO Life Technologies.

### Plasmids and siRNAs

Human ORF cDNA clones were acquired from Thermo Fisher Scientific. siRNAs targeting *PSMC3IP* and *LUCIFERASE* (non-targeting control siRNA) gene expression were purchased from Life Technologies. *XIAP* derived siRNA II was from Cell Signaling Technology and *EPSTI1* derived siRNA was designed as described before [17].

### Cell transfection and treatment

For expression of Myc-tagged fusion proteins, cDNA clones were subcloned into pDEST-Myc-tagged vector using the Gateway cloning system (Life Technologies). Cell transfection using X-tremeGENE 9 (Roche) was performed according to the manufacturer’s instructions. After 24 hours of cell transfection, cells were treated for another 24 hours with recombinant human TRAIL/Apo2 ligand (PeproTech) at final concentration of 80ng/ml in MDA-MB-231 and 100ng/ml in MCF-7.

For RNA interference-mediated gene silencing, cells were seeded and exposed to 50 nM of either gene-specific siRNA or non-targeting control siRNA (siLUC), using Lipofectamine RNAiMAX transfection reagent (Life Technologies) for 48 h. For *EPSTI1* silencing, IFN-α (Chemicon, Millipore) was added to a final concentration of 1000 U/ml 8 hours before harvesting the cells. TRAIL treatment was conducted in the same conditions as in overexpression assays (see above).

### Caspase-8 and caspase-3 activity assays

The activities of caspase 3 and 8 were measured using APOPCYTO Caspase-3 Colorimetric Assay Kit (Medical and Biological Laboratories) and Caspase-8 Colorimetric Assay Kit (BioVision), respectively. Briefly, total cell protein was extracted using ice-cold cell lysis buffer. Then, 100–200 μg of total protein was diluted in 50 μl of lysis buffer and 50 μl of 2× reaction buffer containing 10 mM DTT. 5 μl of caspase 3 or 8 substrate were added into each well of a 96-well microplate. After incubation at 37°C for 3 hours, the absorbance was measured at 405 nm.

### Immunoblot analysis

Protein concentration was determined using the Bio-Rad D_C_ protein assay (Bio-Rad Laboratories). 20 µg of total protein were electrophoresed in 8% SDS-PAGE gel and transferred to Immobilon-P Membrane, PVDF (Millipore). Membranes were incubated overnight at 4°C with primary antibodies. Rabbit polyclonal anti-PSMC3IP, Sigma-Aldrich (HPA044439) at dilution (1:1000), rabbit polyclonal anti-EPSTI1, Sigma Aldrich (SAB2100696) at dilution (1:2000), both from Sigma Aldrich, rabbit polyclonal anti-PARP, Cell Signaling Technology (#9542) at dilution (1:1000) and mouse monoclonal anti-β-actin, Abcam (ab20272), at dilution (1:20000) followed by incubation with the appropriated HRP-conjugated secondary antibody plus enhanced chemiluminescence substrate (GE Healthcare). Protein band amounts were roughly quantified by densitometry using ImageJ (http://rsb.info.nih.gov/ij/), following standard procedures. Loading control protein β-actin was used as a reference to compare relative protein amounts.

### Propidium iodide staining and flow cytometry analysis

Apoptotic cells were quantified by flow cytometry as previously described [21]. Briefly, cells were washed with PBS, fixed in cold 70% ethanol, and then stained with propidium iodide (Sigma Aldrich) while being treated with RNase (Sigma-Aldrich). Quantitative analysis of sub-G_0_/G_1_ cells was carried out in a CouLter XL cytometer using FlowJo software (Tree Star).

### TUNEL assay

Apoptosis was assayed in cell culture using a commercially available kit (In Situ Cell Death Detection kit, fluorescein; Roche, Nutley, NJ) designed to detect terminal deoxynucleotidyl transferase (TdT)–mediated nick end labeling (TUNEL). Cells were fixed in 4% paraformaldehyde and permeabilized in 0.1% Triton X-100 in 0.1% sodium citrate. After washing in PBS, cells were incubated in TUNEL reaction mixture at 37°C for 60 minutes, washed, and mounted. Nuclei were counterstained with Hoechst 33342 Trihydrochloride, trihidrate (Invitrogen).

### Cell viability assay

Cell viability was determined by adding a final concentration of 1.1 mg/mL MTT (3-(4,5-dimethylthiazol-2-yl)-2,5-diphenyltetrazolium bromide (Sigma Aldrich) into the cell culture. Cells were incubated for 2 hours at 37°C, then the medium was removed, MTT was dissolved in DMSO (Sigma Aldrich) and its absorbance was determined at 570nm.

### Correlation in gene expression profiles

We used the microarray data from [22], a compendium of gene expression profiles from 73 normal tissue and cell types, and the protein data from the Human Protein Atlas [23]. We applied a mixture model in order to obtain correlation coefficients that are robust under the presence of noise. We fit the model using the Expectation-Maximization (EM) algorithm [24]. We defined two genes as co-expressed if their EM correlation coefficient was greater than 0.5 and the probability of noise less than 0.5.

### Statistical analysis

Results are expressed as the mean ± standard deviation (SD) of three independent experiments. Statistically significant differences were determined by one-way ANOVA followed by Tukey-Kramer post-test to identify pair wise differences. Differences were considered significant at P < 0.05*. Statistical analyses were carried out using GraphPad Prism (GraphPad Software Inc V4.03, San Diego, CA, USA) software.

## Results and Discussion

### PSMC3IP and EPSTI1 interact with key apoptotic proteins

PSMC3IP and EPSTI1 present relevant transcriptomics/genomics aberrations in breast cancer [7, 11, 15–18], although the molecular mechanisms are still unknown. Since cell proliferation and apoptosis are key for the development of breast tumorigenesis, we sought to explore the eventual implication of these genes in the regulation of these processes. To this end, we exploited the high interconnectivity observed among disease genes [25] to reveal novel direct relationships between well-established BC genes and PSMC3IP and EPSTI1, which could provide a molecular rationale for the implication of these genes in the disease.

An important factor when performing interaction discovery strategies is the selection of the core genes. We extensively mined the literature and the OMIM database [26] to select the most relevant BC genes that are involved in apoptosis or cell proliferation, ending up with 9 BC-apoptosis genes to be included in our interaction screen: AKT1, BAG4, BCAR3, CASP8, CDKN2A, CKN2C, CHEK2, IGF1R and PARP1 (See S1 Table).

We then performed pairwise yeast two-hybrid (Y2H) assays to identify novel interactions between BC-apoptosis genes and PSMC3IP and EPSTI1. Among the 7 high-confidence interactions we identified (see Methods and S1 Table), those of PSMC3IP and EPSTI1 with central proteins in the apoptosis extrinsic pathway, i.e. caspase 8, appeared as the most promising ones. Interestingly, PSMC3IP and EPSTI1 both show a strong co-expression profile with CASP8 in breast normal and cancer tissues, also reflected in higher protein levels (see Methods and S1 Table for further details). In addition, PSMC3IP and EPSTI1 also interact with breast cancer anti-estrogen resistance 3 (BCAR3), which is known to regulate proliferation and induce anti-estrogen resistance in ZR75–1 and MCF-7 breast cancer cells [27]. Finally, we also found EPSTI1 to interact with AKT1 (unpublished data), an antiapoptotic protein that in response to specific stimuli phosphorylates and inactivates certain components of the apoptotic machinery, such as the Bcl2 antagonist of cell death (BAD) and caspase-9 [28, 29]. In breast cancer, the PI3K-Akt pathway is a critical downstream effector of growth factor receptors such as HER2/ErbB2, insulin-like growth factor receptor (IGFR) and epidermal growth factor receptor (EGFR) [30–33]. Taken together, these findings suggest that PSMC3IP and EPSTI1 could play a role in the regulation of the apoptotic response.

### Modulation of PSMC3IP and EPSTI1 expression in breast cancer cells

**Figure 1:**
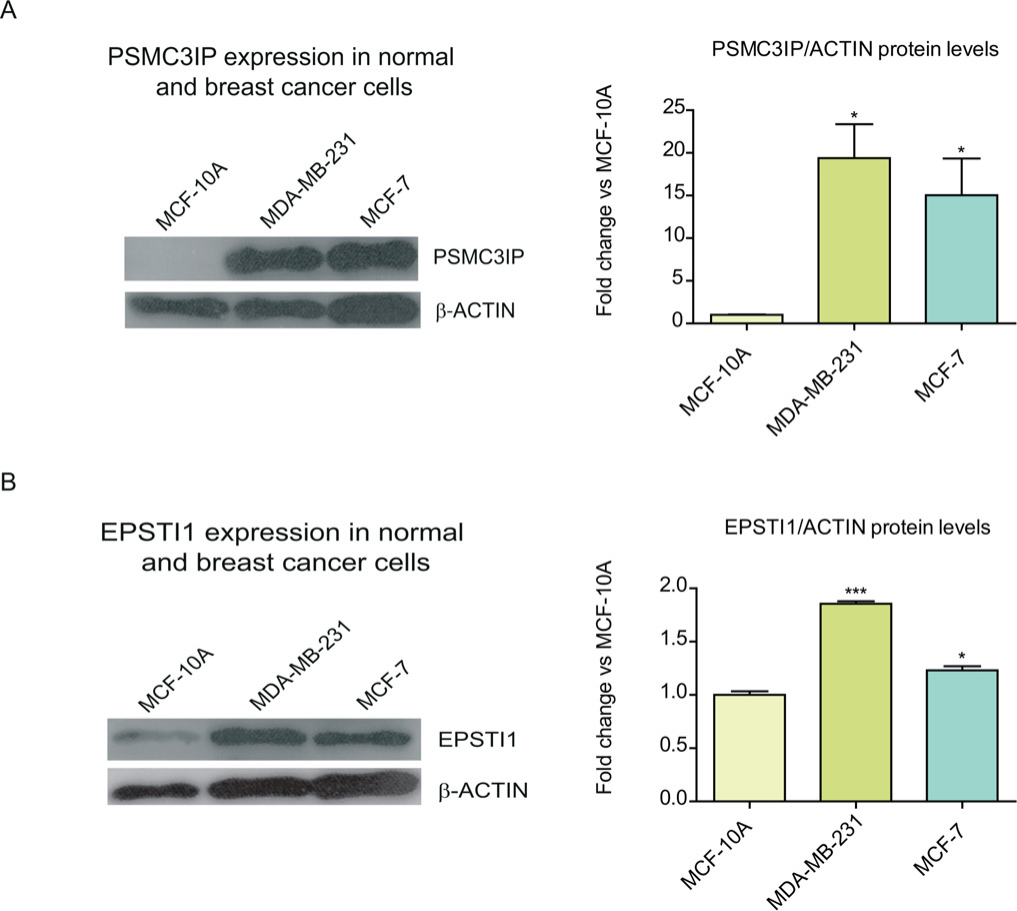
Expression of PSMC3IP and EPSTI1 in normal and breast cancer cell lines. We inspected the endogenous expression of PSMC3IP **(A)** and EPSTI1 **(B)** in two types of breast cancer cell lines, MDA-MB-231 and MCF-7, as compared to a normal breast epithelial cell line, MCF-10A. Estimated protein levels based on densitometry (right) of the immunoblots (left) show a PSMC3IP 19- and 15-fold expression in MDA-MB-231 and MCF-7 cells, while EPSTI1 only shows 1.9- and 1.3-fold in each cell line, respectively. Protein levels were normalized based on the loading control protein β-actin. (*P <0.05, **P<0.01, ***P <0.001 vs MCF-10A cells).

We first investigated whether PSMC3IP and EPSTI1 endogenous protein levels are indeed up-regulated in carcinoma cells such as MCF-7 and MDA-MB-231 (Fig. 1), which represent common breast cancer subtypes differently graded upon hormone dependency and aggressiveness. MCF-7 is a weakly invasive luminal cell line, representative of estrogen receptor (ER)-positive tumors [34]. On the other hand, MDA-MB-231 is a highly invasive basal cell line, and it is often used as model for ER-negative tumors [35]. PSMC3IP is highly overexpressed in both MDA-MB-231 and MCF-7, respectively, compared to the non-tumorigenic breast cancer cell line (MCF-10A) (Fig. 1A). Conversely, EPSTI1 shows a more moderate increase, particularly in MCF-7 cells (1.3-fold, Fig. 1B), as previously observed [17], providing evidence about the heteroclonal nature of MCF-7 sublines [36].

**Figure 2:**
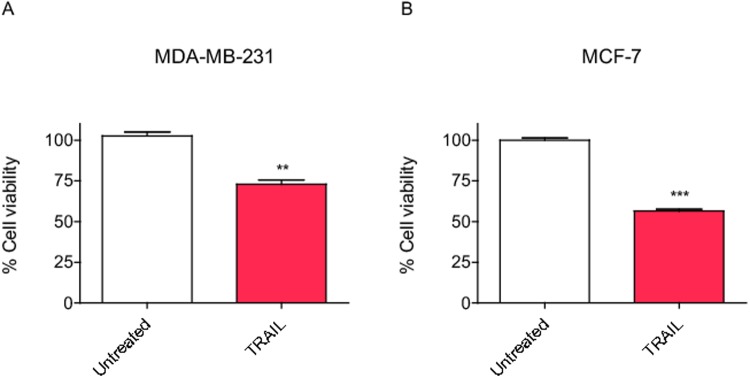
TRAIL-induced apoptosis in breast cancer cells. **(A)** MDA-MB-231 cells treated with the apoptosis inducing ligand TRAIL at 80ng/mL for 24h show a moderate decrease in cell viability while **(B)** MCF-7 cells treated with TRAIL at 100ng/mL.for 24h show a more pronounced decrease in viability. Each bar represents the mean ±SD of three experiments performed in duplicate (*P <0.05, **P <0.01, ***P <0.001 vs untreated cells).

Furthermore, to enhance the phenotypic response that PSMC3IP and EPSTI1 might have on caspase-8, we induced the extrinsic apoptotic pathway using TRAIL, which typically generated about 25 and 35% cell viability decrease in non-transfected MDA-MB-231 and MCF-7 cells, respectively (Fig. 2), in agreement with the data reported in the literature [37–39].

**Figure 3:**
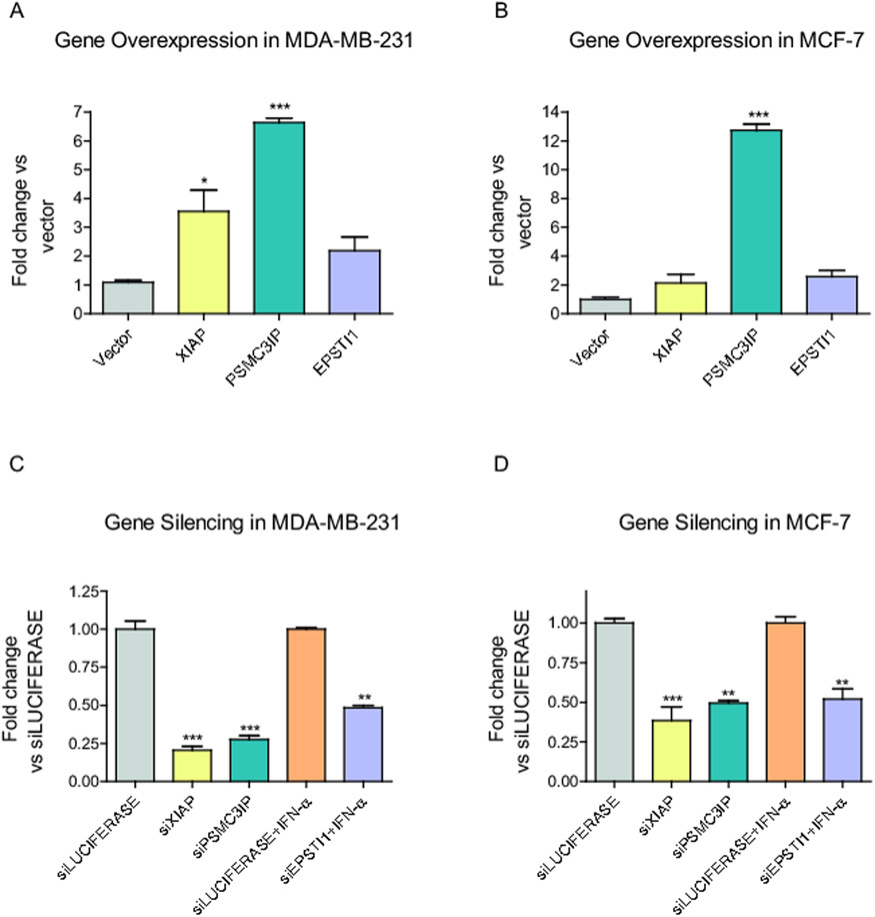
Modulation of PSMC3IP and EPSTI1 expression in breast cancer cells. **(A-B)** Genes were overexpressed as Myc-tagged fusion proteins in different cell lines and protein relative levels were analysed based on MYC-tag empty transfection vector (Vector). **(C-D)** Endogenous gene expression was silenced using specific siRNA and depletion levels were analysed based on siRNA against luciferase expression (siLUC) as a negative control. Prior to depletion experiments, EPSTI1 expression was induced by treating cells with IFN-α at 1000 U/ml for 8h. XIAP was used as a reference anti-apoptotic protein in all experiments. Each bar represents the mean ±SD of three experiments performed in duplicate (*P <0.05, **P <0.01, ***P <0.001 vs MYC-tag vector in overexpression assays and vs siLUCIFERASE in silencing).

We then modulated PSMC3IP and EPSTI1 differential expression by either cDNA or siRNA knockout cell transfection. We included the X-linked inhibitor of apoptosis protein (XIAP) as an anti-apoptotic reference gene, since it is a well-characterized inhibitor of caspase-3, caspase-7 and caspase-9 [40, 41] (Fig. 3). Compared to cells transfected with empty vectors, we observed a highly overexpression of PSMC3IP in both cell lines (MDA-MB-231, 6.5-fold; MCF-7, 13-fold) (Fig. 3A–3B), albeit we only achieved a moderate EPSTI1 overexpression (2.1-fold and 2.6-fold, respectively (Fig. 3A–3B). On the other hand, by siRNA transfection, we were able to reduce PSMC3IP levels by 70% in MDA-MB-231 and 50% in MCF-7 cells compared to cells transfected with control siRNA (siLUC) (Fig. 3C–3D). To maximize and visualize the effect of EPSTI1 depletion, we induced its endogenous expression with IFN-α prior to gene silencing in both cell lines (Fig. 3C–3D), as previously reported [17].

We subsequently performed a variety of functional assays to measure the apoptotic activity of the two candidate genes in BC cells under basal or apoptotic induced conditions.

### PSMC3IP and EPSTI1 expression regulates caspase-8 activity

**Figure 4:**
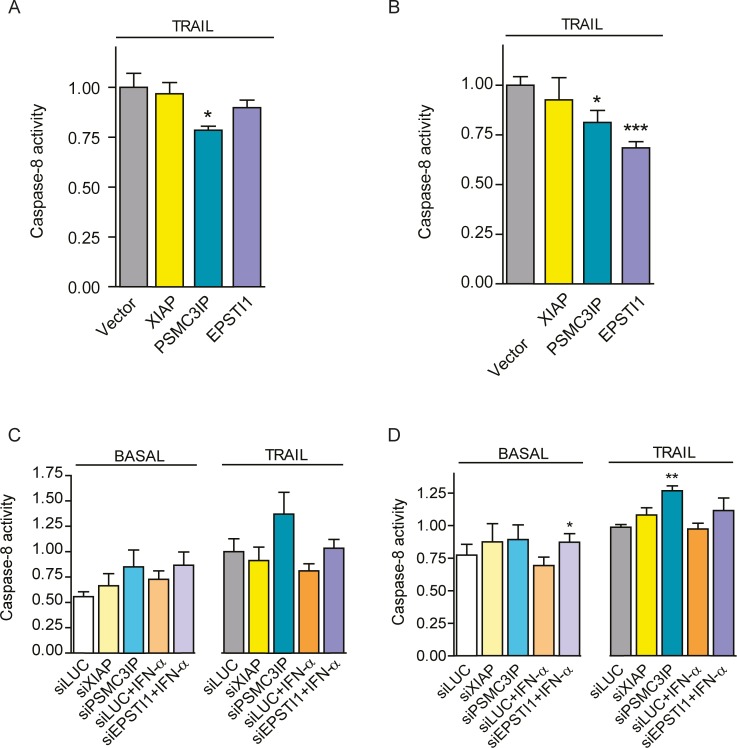
Caspase-8 activity modulation. Caspase-8 activity was quantified by measuring the chromophore levels released from caspase-8 cleaved substrates. Overexpression of PSMC3IP or EPSTI1 in TRAIL-treated MDA-MB-231 **(A)** and MCF-7 cells **(B)** decrease caspase-8 activity based on the MYC-tag empty transfection vector (Vector) as control. Caspase-8 activity was also measured after gene silencing in MDA-MB-231 **(C)** and MCF-7 **(D)** cells, under basal or TRAIL-treated conditions. Genes were silenced using specific siRNAs targeting *XIAP*, *PSMC3IP* or *EPSTI1* and siRNA against luciferase expression (siLUC) was used as a negative control. *EPSTI1*-depleted cells were previously treated with IFN-α at 1000 U/ml for 8h. MDA-MB-231 and MCF-7 cells were treated with TRAIL for 24h at 80 or 100ng/mL, respectively. XIAP was used as an anti-apoptotic reference in all experiments. Each bar represents the mean ±SD of three experiments performed in duplicate (*P <0.05, **P <0.01, ***P <0.001 vs MYC-tag vector in overexpression assays and vs siLUCIFERASE in silencing).

Since PSMC3IP and EPSTI1 both interact with caspase-8, we first sought to examine the influence of these interactions on caspase-8 activity, which is upstream of the apoptotic cascade. We only observe significant caspase-8 activity decrease upon PSMC3IP overexpression in MDA-MB-231 cells (1.6-fold P<0.05) (Fig. 4A). But interestingly, both individual candidates do decrease caspase-8 activity in MCF-7 cells (PSMC3IP, 1.2-fold P<0.05; EPSTI1, 1.5-fold P<0.001) (Fig. 4B). In agreement with the overexpression results, caspase-8 activity increases after *PSMC3IP* or *EPSTI1* gene silencing in both cell lines (Fig. 4C–D), although preeminently in MCF-7 TRAIL-treated cells upon *PSMC3IP* depletion (1.3-fold, P<0.01) and under basal conditions upon *EPSTI1* depletion (1.3-fold, P<0.05) (Fig. 4D). As expected, XIAP overexpression or silencing does not affect caspase-8 activity, since the inhibitory effect of XIAP is on downstream caspases like caspase-3 or caspase-7.

Collectively, these results indicate that PSMC3IP and EPSTI1 do modulate caspase-8 activity, suggesting their involvement in the extrinsic apoptotic pathway in breast cancer cells.

### PSMC3IP and EPSTI1 expression modulates caspase-3 activity and PARP cleavage

**Figure 5:**
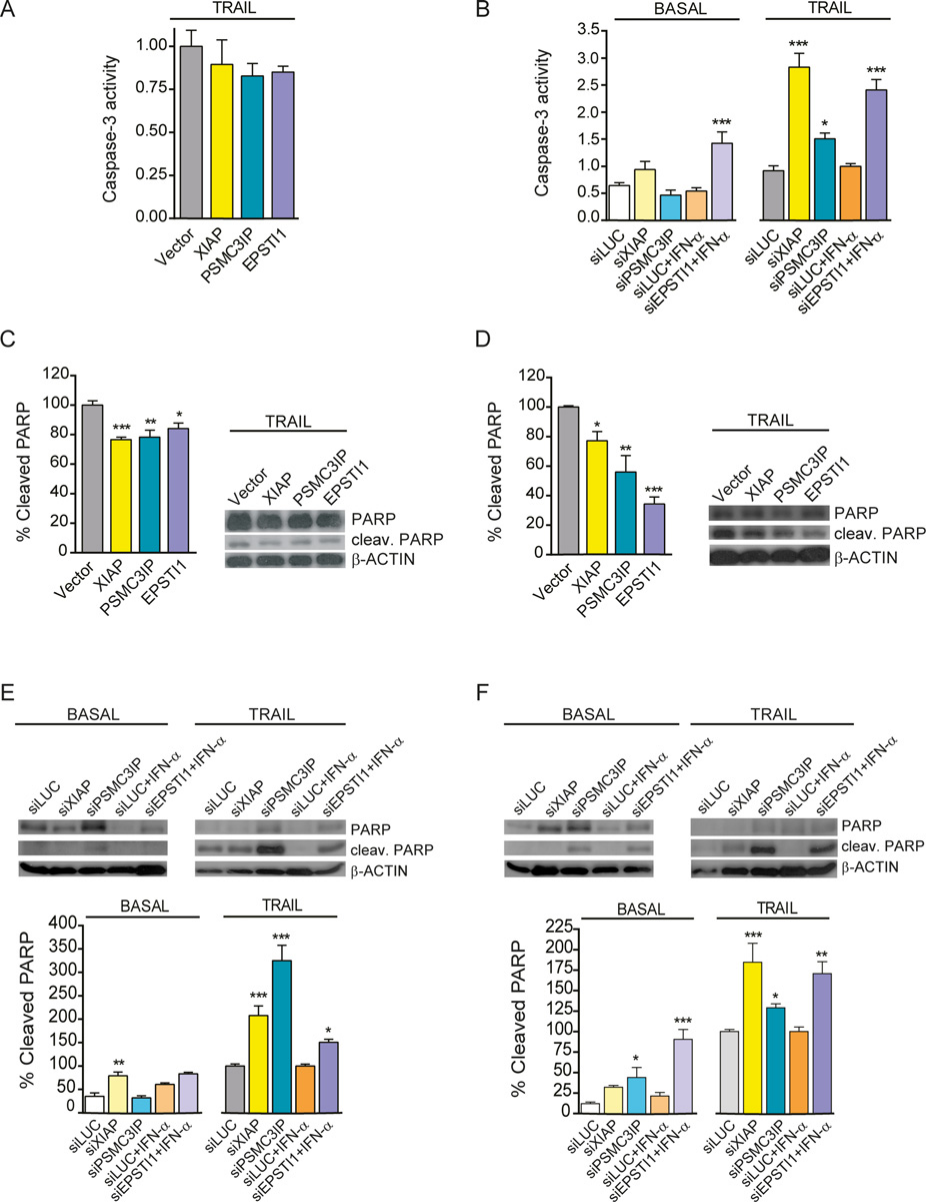
Caspase-3 activity modulation and analysis of cleaved PARP protein levels. **(A)** Caspase-3 activity was measured by colorimetric quantification of fluorescent products released from caspase-3 cleaved substrates.in TRAIL-treated MDA-MB-231 cells overexpressing XIAP, PSMC3IP or EPSTI1. None of the overexpressed genes was able to significantly decrease the activity relative to MYC-tag empty transfection vector (Vector) as control. **(B)** Caspase-3 activity was also measured in MDA-MB-231 cells under basal or TRAIL-treated conditions after gene silencing using specific siRNA targeting *XIAP*, *PSMC3IP* or *EPSTI1*. **(C)** Immunoblot analysis of cleaved PARP protein levels in gene-overexpressing MDA-MB-231 cells under TRAIL conditions, using MYC-tag transfection vector (Vector) as a negative control, reveals an attenuation of downstream apoptotic cascades. **(D)** This effect is much more pronounced in TRAIL-treated MCF-7 cells. **(E)** Analysis of cleaved PARP protein levels after gene silencing in MDA-MB-231 cells. siLUC was used as a negative control. **(F)** The same analysis in MCF-7 cells shows a more pronounced effect, even under basal conditions. *EPSTI1*-depleted cells were previously treated with IFN-α at 1000 U/ml for 8h. In apoptosis-induced conditions, MDA-MB-231 and MCF-7 cells were treated with TRAIL for 24h at 80 or 100ng/mL, respectively. XIAP was used as an anti-apoptotic reference in all experiments. Each bar represents the mean ±SD of three experiments performed in duplicate (*P <0.05, **P <0.01, ***P <0.001 vs MYC-tag vector in overexpression assays and vs siLUCIFERASE in silencing).

It is well known that the activation of initiator caspases, like caspase-8, leads to the activation of the executioner caspases, such as caspase-3 in MDA-MB-231 cells [42]. Therefore, we investigated whether PSMC3IP or EPSTI1 expression affects caspase-3 activity under basal or apoptotic conditions in MDA-MB-231 cells. We observed that overexpression of either gene does not alter caspase-3 activity levels (Fig. 5A). Yet, *EPSTI1* silencing results in an increased caspase-3 activity in both basal conditions (2.6-fold, P<0.001) and upon TRAIL treatment (2.4-fold, P<0.001), giving similar results as the silencing of the anti-apoptotic gene XIAP. On other hand, *PSMC3IP* silencing is only able to increase caspase-3 activity under TRAIL treatment (1.5-fold, P<0.05) (Fig. 5B). These results indicate that indeed EPSTI1 and PSMC3IP modulate caspase-3 activity in MDA-MB.231 cells, albeit at varying degrees of apoptotic stimulation.

Caspase-7 and caspase-3 coordinate the last phase of apoptosis by cleaving protein substrates such as PARP [43, 44], which has an essential role in repairing single-strand breaks (SSBs). PARP is inactivated by caspase cleavage, causing SSB repair inhibition that can result in lethal DNA damage [45]. Interestingly, overexpressing PSMC3IP or EPSTI1 show a significant decrease in PARP cleavage in MDA-MB-231 and MCF-7 cells (1.3-fold, P<0.01; 1.8-fold, P<0.01) (1.2-fold, P<0.05; 2.9-fold, P<0.001) (Fig. 5C–D). Furthermore, in agreement with our caspase-3 activity results, there is an increase of cleaved PARP levels after *PSMC3IP* or *EPSTI1* gene depletion in MDA-MB-231 cells upon TRAIL induction (PSMC3IP, 3.2-fold, P<0.001; EPSTI1, 1.5-fold, P<0.05) (Fig. 5E). This effect is more pronounced in MCF-7 cells, where PARP cleavage is increased even in basal conditions (EPSTI1, 4.2-fold, P<0.001; PSMC3IP, 3.6-fold, P<0.05) (Fig. 5F). Taken together, although preclinical studies have shown that ER-negative breast cancer cell lines are more sensitive to PARP inhibitors compared to luminal cells [46], our results indicate that PARP cleavage is similarly affected by the expression of putative extrinsic regulators such as PSMC3IP and EPSTI1 in both ER-negative (i.e. MDA-MB-231) and in luminal breast cancer cell line (i.e. MCF-7). In addition, since EPSTI1 also interacts with AKT1, it could play an alternative role in modulating apoptosis through PI3K pathway, in line with preclinical data that demonstrate synergistic activity when PARP inhibitors are combined with PI3K inhibitors [47].

### Increased DNA fragmentation and reduced cell viability are associated with PSMC3IP and EPSTI1 down-regulation

**Figure 6:**
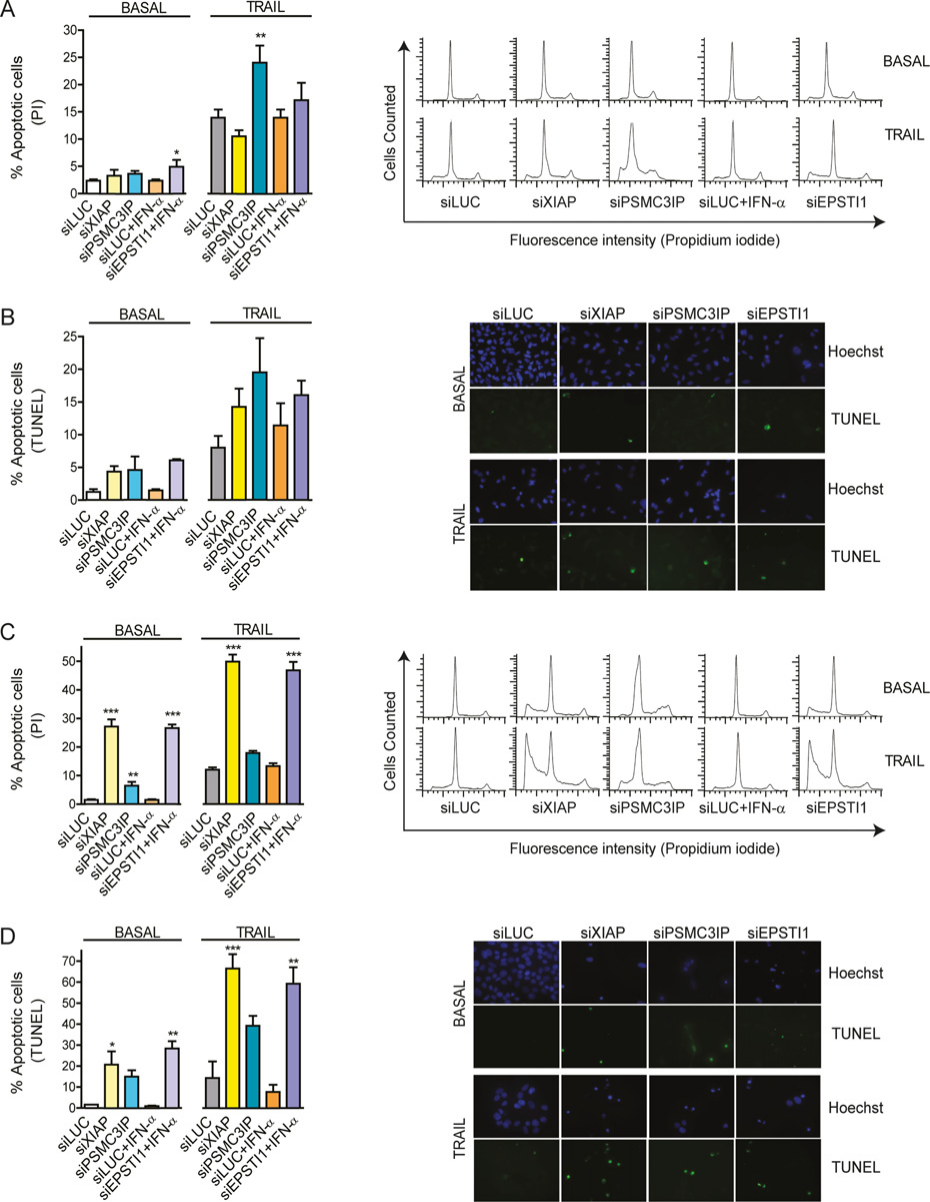
Detection of DNA fragmentation. **(A)** The number of apoptotic MDA-MB-231 cells was quantified by flow cytometry using propidium iodide DNA staining after gene depletion under basal or TRAIL-treated conditions (i.e. measurement of the sub-G0/G1 peak in the fluorescence DNA histograms, *right panels*). **(B)** Apoptosis was also evaluated in MDA-MB-231 by the inspection of DNA fragmentation by TUNEL (fluorescein-12-dUTP labeled fragmented DNA) staining (*right panels*). Cell nuclei were stained with Hoechst (blue fluorescence) to estimate the number of total cells. (**C**) A similar analysis was carried out in MCF-7 cells. The measurement of the sub-G0/G1 peaks (*right panels*) indicates a significantly higher number of apoptotic gene-depleted cells even under basal conditions (*left*). (**D**) TUNEL positive nuclei displaying green fluorescence are observed under basal conditions (*right*), although a higher number is clearly observed under TRAIL-induced conditions (*left*). siLUC was used as a negative control, XIAP was used as an anti-apoptotic reference in all experiments. *EPSTI1*-depleted cells were previously treated with IFN-α at 1000 U/ml for 8h. In apoptosis-induced conditions, cells were treated with TRAIL for 24h, at 80 or 100ng/mL respectively. Each bar represents the mean ±SD of three experiments performed in duplicate (*P <0.05, **P <0.01, ***P <0.001 vs siLUC).

DNA fragmentation, resulting from apoptotic signalling cascades, is a hallmark of late-stage apoptosis [48]. Hence, we wanted to examine whether EPSTI1 and PSMC3IP are able to alter the final apoptotic response beyond the modification of caspase activity. To this end, we quantified the number of apoptotic cells by flow cytometry (i.e. measurement of the sub-G_0_/G_1_ peak in the fluorescence histograms) and we further examined DNA fragmentation by TUNEL assays (Fig. 6).

In MDA-MB-231 cells, we detect an increased number of apoptotic cells upon *PSMC3IP* silencing under TRAIL conditions (1.6-fold, P<0.01), while *EPSTI1* depletion has already a similar effect under basal conditions (1.7-fold, P<0.05) (Fig. 6A). Yet, TUNEL-based fluorescent microscopy images do not show conclusive results (Fig. 6B). Interestingly, MCF-7 cells show a clearer phenotype, where *PSMC3IP* depletion is able to increase the number of apoptotic cells in basal conditions (4.3-fold, P<0.01) (Fig. 6C) and *EPSTI1* silencing is now able to induce a high increase of apoptotic cells under both conditions (17.7-fold, P<0.001; 3.5-fold, P<0.001) (Fig. 6C). These findings are in accordance with apoptotic positive cells observed in TUNEL assays (Fig. 6D).

**Figure 7:**
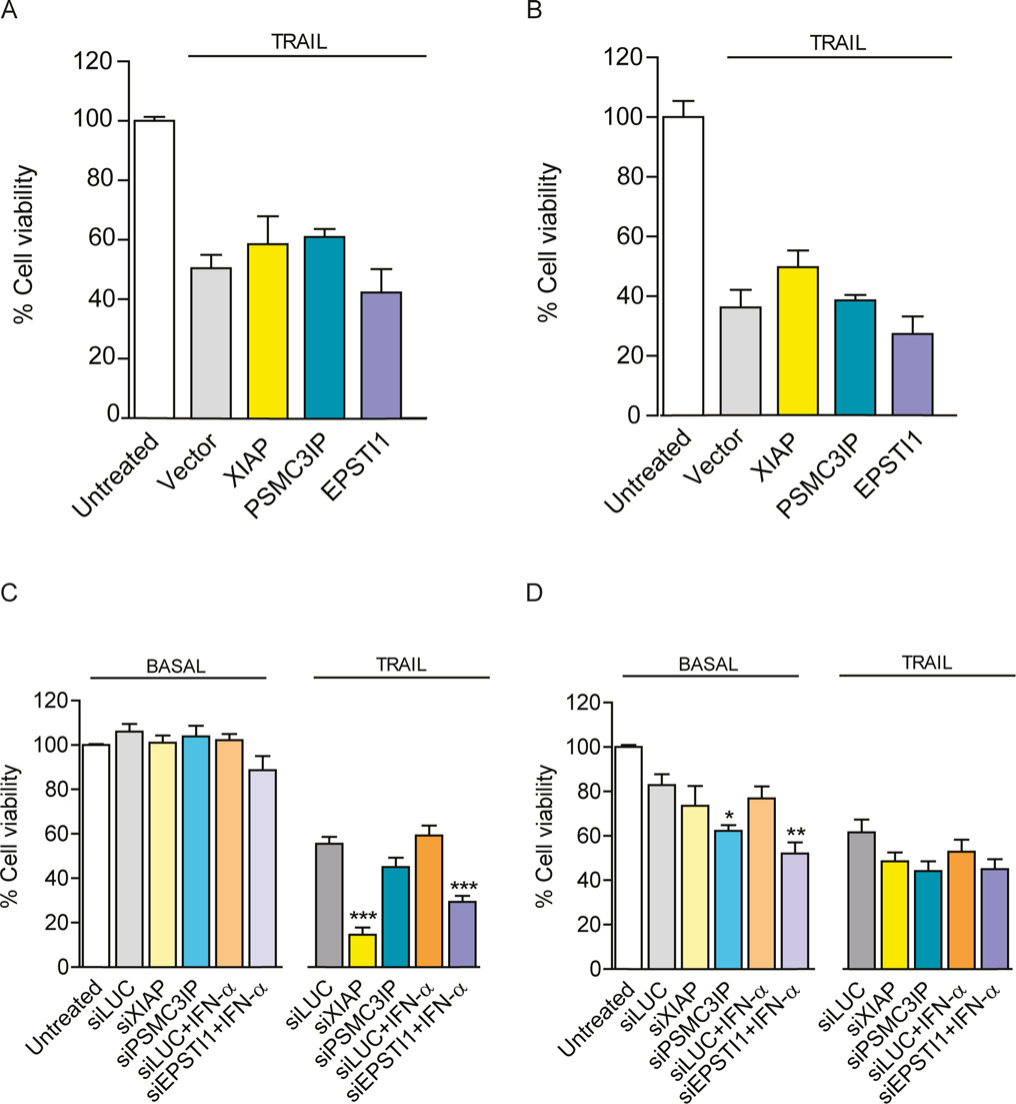
Cell viability and recovery. Cell viability was determined by MTT absorbance assays **(A)** Histograms showing the viability of PSMC3IP or EPSTI1-overexpressing MDA-MB-231 cells and **(B)** MCF-7 cells under TRAIL-induced conditions. Based on empty vector (MYC-tag) as a negative control, we do not observe a significant recovery of apoptosis-induced cells after gene overexpression. **(C)** Viability measurement of gene-depleted MDA-MB-231 cells reveals that EPSTI1 depletion reduces about 50% of viability as compared to siLUC negative control. **(D)** Intriguingly, in MCF-7 cells, both PSMC3IP and EPSTI1 depletion lead to a decreased viability under basal but not under TRAIL-treated conditions. XIAP was used as an anti-apoptotic reference in all experiments. *EPSTI1*-depleted cells were previously treated with IFN-α at 1000 U/ml for 8h. In apoptosis-induced conditions, cells were treated with TRAIL for 24h, at 80 or 100ng/mL respectively. Each bar represents the mean ±SD of three experiments performed in duplicate (*P <0.05, **P <0.01, ***P <0.001 vs siLUC).

Lastly, we sought to determine breast cancer cells viability after PSMC3IP or EPSTI1 expression modulation. As observed in Fig. 7, up-regulation of either gene is not able to recapitulate the viability of neither TRAIL-treated MDA-MB-231 (Fig. 7A) nor MCF-7 cells (Fig. 7B). Conversely, *EPSTI1* down-regulation does decrease the viability of MDA-MB-231 cells treated with TRAIL (1.9-fold, P<0.001) (Fig. 7C). Intriguingly, *PSMC3IP* or *EPSTI1* silencing only induces significant MCF-7 cell viability decrease in basal conditions (1.3-fold, P<0.05 and 1.5-fold, P<0.01 respectively) (Fig. 7D).

## Concluding Remarks

**Figure 8:**
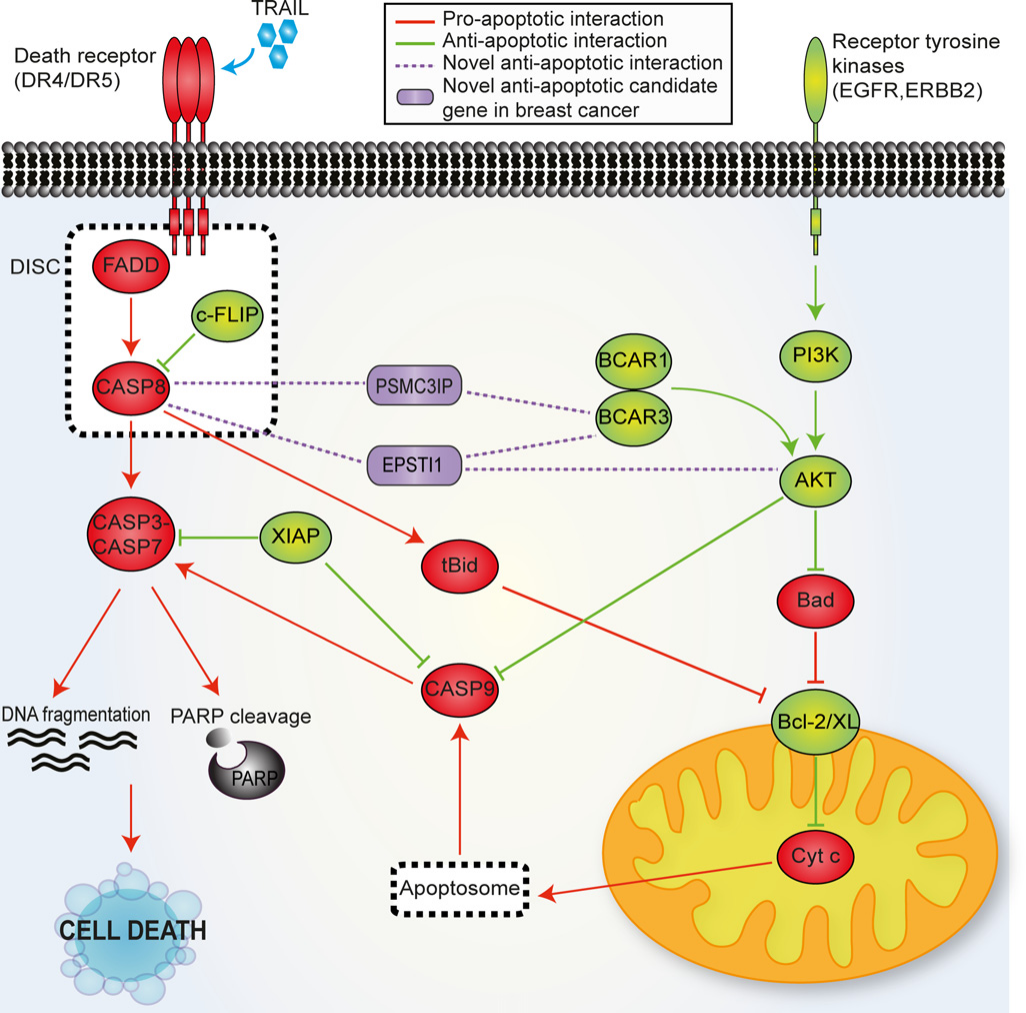
Mechanistic model of PSMC3IP and EPSTI1 as putative apoptotic factors. The extrinsic apoptosis pathway is initiated by the ligand binding to the death receptor, forming the DISC complex and leading to the activation of the caspase signaling cascade. The overexpression of EGFR and HER2 or activation of components of their downstream signaling pathways (i.e. PI3K pathway) induce an anti-apoptotic signaling through PI3K-Akt pathway in endocrine resistant breast cancer cells. Based on our findings, we suggest that PSMC3IP and EPSTI1 may regulate the apoptotic pathway via the physical interaction mainly with the apoptosis initiator CASP8, but also with AKT1 and BCAR3. Pro-apoptotic proteins are displayed in red and anti-apoptotic in green. Candidate proteins are displayed in purple.

As shown in the summary Tables 1 and 2, collectively, our findings reveal that PSMC3IP and EPSTI1 have a strong anti-apoptotic role in breast cancer cells, particularly in estrogen receptor positive and triple negative, by means of physical interaction with the apoptosis initiator caspase-8 (Fig. 8). Interestingly, PSCM3IP or EPSTI1 depletion in breast cancer cells show increased DNA fragmentation and reduced cell viability even in the absence of apoptotic stimuli, indicating that they might also modulate the apoptotic pathway trough alternative mechanisms, such as by BCAR3 or AKT1 interaction, particularly in case of EPSTI1. Although further studies are required to gain deeper insight into the molecular mechanisms underlying the anti-apoptotic role of PSMC3IP and EPSTI1 in breast cancer, our findings highlight them beforehand as very interesting therapeutic targets, preeminently for their ability to apoptosis sensitization.

**Table 1:** Overview of the results obtained from apoptosis-related functional assays upon modulation of PSMC3IP expression in breast cancer cells.

**PSMC3IP**	**BASAL**		**TRAIL**			
	**MDA-MB-231 silencing**	**MCF-7 silencing**	**MDA-MB-231 overexpression**	**MDA-MB-231 silencing**	**MCF-7 overexpression**	**MCF-7 silencing**
**caspase 8 activity**	ns	ns	-	ns	-	**+ +**
**caspase 3 activity**	ns	na	ns	**+**	na	na
**cleaved PARP**	ns	**+**	--	**+ + +**	--	**+**
**apoptotic cells**	ns	**+ +**	nd	**+ +**	nd	ns
**cell viability**	ns	-	ns	ns	ns	ns

**Table 2:** Overview of the results obtained from apoptosis-related functional assays upon modulation of EPSTI1 expression in breast cancer cells.

**EPSTI1**	**BASAL**		**TRAIL**			
	**MDA-MB-231 silencing**	**MCF-7 silencing**	**MDA-MB-231 overexpression**	**MDA-MB-231 silencing**	**MCF-7 overexpression**	**MCF-7 silencing**
**caspase 8 activity**	ns	**+**	ns	ns	---	ns
**caspase 3 activity**	**+ + +**	na	ns	**+ + +**	na	na
**cleaved PARP**	ns	**+ + +**	-	**+**	---	**+ +**
**apoptotic cells**	**+**	**+ + +**	nd	ns	nd	**+ + +**
**cell viability**	ns	--	ns	---	ns	ns

Statistically significant increase or decrease is highlighted +*P* <0.05, ++*P* <0.01, +++*P* <0.001 and - *P* <0.05, - - *P* <0.01, - - - *P* <0.001 respectively.

nd = no data available, na = not applicable, ns = no significant differences.

## Supporting Information

S1 Table PSMC3IP and EPSTI1 interactions with BC-apoptosis genes. List of selected BC-apoptosis genes and detected interactions with PSMC3IP and EPSTI1 observed by matrix Y2H screens. Candidate gene expression in healthy and carcinoma breast tissues and their co-expression with core BC-apoptotic genes.
(XLSX)
